# IL-33-mediated activation of mast cells is involved in the progression of imiquimod-induced psoriasis-like dermatitis

**DOI:** 10.1186/s12964-023-01075-7

**Published:** 2023-03-09

**Authors:** Xuyue Zhou, Yu Hu, Lingxi Liu, Lihao Liu, Hongying Chen, Dan Huang, Mei Ju, Chao Luan, Kun Chen, Jiaan Zhang

**Affiliations:** Institute of Dermatology, Chinese Academy of Medical Science and Peking Union Medical College, Nanjing, China

## Abstract

**Background:**

Psoriasis is a chronic inflammatory dermatosis with an unclear pathogenesis. Mast cells (MCs) can serve as a bridge between innate and adaptive immunity and are involved in the regulation of the inflammatory state and immune homeostasis in diseases. MCs constitutively express interleukin-33 receptor T1/ST2 (IL-33R). IL-33 is a potent MCs activator that is actively secreted by keratinocytes in psoriasis. However, the regulatory role of MCs in psoriasis remains uncertain. Therefore, we hypothesised that IL-33 could promote MC activation to regulate psoriasis development.

**Methods:**

We performed experiments on wild-type (WT) and MC-deficient (Kit Wsh/Wsh) mice, established psoriasis-like mouse models using imiquimod (IMQ), and performed RNA sequencing and transcriptomic analysis of skin lesions. Exogenous administration was performed using recombinant IL-33. Validation and evaluation were performed using PSI scoring, immunofluorescence, immunohistochemistry, and qPCR.

**Results:**

We observed an upregulation in the number and activation of MCs in patients with psoriasis and in IMQ-induced psoriasis-like dermatitis. Deficiency of MCs ameliorates IMQ-induced psoriatic dermatitis at an early stage. IL-33 is increased and co-localized with MCs in the dermis of psoriasis-like lesions using immunofluorescence. Compared to WT mice, IMQ-induced Kit^Wsh/Wsh^ mice demonstrated a delayed response to exogenous IL-33.

**Conclusions:**

MCs are activated by IL-33 in the early stages of psoriasis and exacerbate psoriasis-associated skin inflammation. The regulation of MC homeostasis may be a potential therapeutic strategy for psoriasis.

**Video Abstract**

**Supplementary Information:**

The online version contains supplementary material available at 10.1186/s12964-023-01075-7.

## Introduction

Psoriasis, one of the most common inflammatory immune dermatological diseases worldwide, is characterised by over-proliferation of keratinocytes and abnormal infiltration of immune cells in the dermis and epidermis. The disease pathogenesis is related to multiple cytokine-mediated crosstalk and to the dysfunction between the cutaneous innate immune system, adaptive immune cells, and resident cutaneous cells [1]. Infiltration of CD4^+^ and CD8^+^ T cells, mast cells (MCs), neutrophils, natural killer cells, natural killer T cells, macrophages, and innate lymphocytes into the dermis and epidermis is a hallmark of the disease [2]. Mast cells, a crucial component of the psoriatic skin microenvironment, are considered the first responders that initiate and amplify the immune response; therefore, activation of mast cells may be critical at an early stage of psoriasis.

MCs, being local tissue sentinels, are primarily located in barrier tissues such as the skin, respiratory tract, and intestinal mucosa. Under physiological conditions, MCs are located in the dermis, in close proximity to blood vessels, nerves, and hair follicles. Upon activation, via external environmental stimuli or pathogen invasion, MCs release a variety of mediators, including cytokines and chemokines synthesised de novo, histamine, proteases, leukotrienes, and prostaglandins pre-stored in cytoplasmic granules [3]. In several diseases, MCs play a dual role in inflammation and immune regulation. Corneal injury leads to increased release of IL-33 from epithelial cells, and this activates MCs to secrete the neutrophil chemokine CXCL2 and initiate early neutrophil recruitment [4]. In contrast, MCs prevent acute ischemic kidney injury through the release of MC protease 4, which limits excessive neutrophil activation and recruitment [5]. During colorectal cancer (CRC) development, MCs also play a dual role through their interaction with tumour-infiltrating lymphocytes. On one hand, MCs promote colitis-associated CRC by suppressing CD8^+^ cell density, and on the other hand, MCs exert a protective role in colitis-independent sporadic CRC by increasing CD3^+^ cell density [6]. Nevertheless, the role of MCs in the development of psoriasis remains controversial.

IL-33 is an alarmin that is released in response to infection, injury, or other stress-induced inflammatory conditions. This alarmin exerts a pleiotropic activity in innate and adaptive immune responses, playing a crucial role in tissue homeostasis, infection, inflammation, metabolism, and neoplastic diseases [7, 8]. IL-33 promotes MC activation, survival, and proliferation independently of the classical IgE-Ag cross-linking-induced pathway. MCs constitutively express IL-33 receptor T1/ST2 (IL-33R) on their surface; binding of IL-33 to its receptor induces MCs to produce cytokines, including IL-1β, TNF-α, IL-6, and IL-13 [9, 10]. The relevance of the IL-33/ST2 axis in psoriasis has been highlighted in several studies. In psoriasis, IL-33 is primarily secreted by keratinocytes; however, alterations of serum IL-33 levels in patients with psoriasis and the role of IL-33 in the disease currently demonstrate conflicting results [11–15]. The role of MCs and IL-33 in psoriasis remains unclear. Here, we hypothesise that IL-33 released by keratinocytes in psoriasis may influence disease development through MC activation.

## Materials and methods

### Patients

All experiments conducted were approved by the Institutional Ethical Review Board of Peking Union Medical College (No.2022-KY-036) and in compliance with the Declaration of Helsinki. All participants were fully informed of the details of the study and provided written informed consent. Participants were recruited between January 2022 and September 2022 at the Hospital of Dermatology, Chinese Academy of Medical Sciences, and Peking Union Medical College, Nanjing, China. Patients were diagnosed with psoriasis vulgaris by two senior dermatologists. Approximately 6-mm punch biopsies were taken from the lesioned skin of the patients. Surgical skin waste from healthy individuals who underwent cosmetic surgery was used as negative controls. Additional file 1: Table S1 presents the clinical features of the study participants.

### Animals

C57BL/6 J control mice were purchased from Beijing Vital River Laboratory Animal Technology (Beijing, China). Mast cell deficient-Kit^Wsh/Wsh^ mice were obtained from the Mode Animal Research Center of Nanjing University. Six- to eight-week-old gender-matched mice were used for the described experiments. All mice were housed under specific-pathogen-free conditions, with dark/light cycles of 12 h, constant temperature of 22–25 °C, and humidity of 30–70%, according to the recommendations of the Guide for the Care and Use of Laboratory Animals of the United States National Institute of Health. All experiments were approved by the Institutional Animal Care and Use Committee of the Institute of Dermatology, Chinese Academy of Medical Science (Approval No.2022-DW-006).

### Mouse experiments

For the psoriasis mouse model, the dorsal skin of mice was shaved 2 days prior to the application of the topical treatment. Mice were initially anesthetised with 4% isoflurane until movement arrest was observed, then maintained with a continuous flow of 1% isoflurane in an isoflurane chamber. IMQ cream (5%) (Mingxin Pharmaceuticals, Sichuan, China), 62.5 mg, was topically applied to the shaved 2 cm × 3 cm skin portion for six consecutive days. Control mice were treated with 62.5 mg of Vaseline (Vas) as vehicle control under the same conditions. The first day of IMQ or Vas application was defined as Day 0, and mice were euthanised on Day 4 and Day 6.

For the exogenous IL-33 administration, 1 μg of recombinant murine IL-33 (PeproTech, USA, cat: 210–33) or phosphate-buffered saline (PBS; control) in a final volume of 100 μL were administered daily through intraperitoneal injection, 1 h prior to IMQ treatment.

The severity of skin inflammation was evaluated daily by two independent researchers, using the scoring system of the psoriasis severity index (PSI). This score rated erythema, scaling, and skin thickness on a scale from 0 to 4: 0—none; 1—slight; 2—moderate; 3—marked; and 4—very marked, according to previous studies [16]. A cumulative score was generated from these parameters (scale 0–12). Mice were euthanised and samples harvested for further analyses at the respective time points.

### Histopathological analyses of the skin

The skin samples from human participants and the mouse dorsal skin samples on Day 4 and Day 6 post-modelling, were collected, fixed in 4% paraformaldehyde for 48 h, dehydrated, embedded in paraffin, and cut into 4-μm sections for histological analysis. Skin tissue sections were stained with haematoxylin and eosin (H&E) using standard protocols. The epidermal thickness throughout the section was calculated based on the average of three randomly selected areas from three image fields per mouse. Toluidine blue staining was performed using standard protocols for MC identification. High-power field allowed the counting of MCs, characterised by their deep blue-purple staining, size, and multiple granules. The images were obtained using an inverted light microscope (Olympus), and data quantification was performed using the ImageJ-Pro Plus software under double-blind conditions.

### Immunohistochemistry (IHC) and immunofluorescence staining

For immunohistochemistry, deparaffinised sections were boiled in sodium citrate buffer (pH 6.0) for antigen retrieval. Endogenous peroxidase activity was inhibited by 3% hydrogen peroxide, and nonspecific binding was blocked using bovine serum albumin (BSA). Sections were then incubated overnight with the primary antibodies (Ki67: 1:750, #GB111141, Servicebio; CD31: 1: 600, #GB11063-2, Servicebio) at 4 °C. After incubation, sections were washed and incubated with the secondary antibody, HRP-conjugated goat anti-rabbit IgG (1:200, #G1213, Servicebio) at 23–25 °C for 50 min. Immunostaining was developed using a DAB solution (#G1211, Servicebio).

For immunofluorescence, deparaffinised sections were boiled for antigen retrieval and blocked for non-specific binding with BSA. The sections were then either incubated with fluorescent avidin conjugates (#A21370, Invitrogen) for 1 h at 23–25 °C for the identification of MCs, or overnight with the primary antibody at 4 °C (IL-33: 1:100, #AF3626, R&D Systems). After overnight incubation, sections were washed and incubated with the secondary antibody, Cy3-conjugated donkey anti-goat IgG (1:200, # GB21404, Servicebio), for 1 h at RT. Nuclei were stained with DAPI (#GDP1024, Servicebio). Fluorescence images were captured using a microscope (Nikon; Tokyo, Japan).

### RNA sequencing and analysis

Whole skin samples of C57BL/6 J and Kit^Wsh/Wsh^ mice (IMQ-treated and control) were stored in RNAlater™ solution (#AM7021, Invitrogen). Total RNA was isolated using Trizol (#15,596,018, Invitrogen) according to the manufacturer’s instructions from the twelve samples, three per group, and RNA quality was assessed using an Agilent 2100 Bioanalyzer (Agilent Technologies). The RNA was sheared and reverse transcribed using random primers to obtain cDNA, which was used for library construction. This cDNA was subsequently sequenced using the BGISEQ-500 platform and mapped to the mouse genome. The sequencing data was filtered using SOAPnuke (v1.5.2) [17]. Qualified raw data were provided by the Beijing Genomics Institute (www.genomics.org.cn, BGI) in FASTQ format. Differentially expressed genes (DEGs) were defined as |log2FC|≥ 1.5 and Q value ≤ 0.05. Gene Ontology (GO; http://www.geneontology.org/) and Kyoto Encyclopaedia of Genes and Genomes (KEGG; https://www.kegg.jp/) pathway enrichment analyses were performed.

### RNA isolation and real‐time quantitative PCR

Total RNA from skin samples was extracted using Trizol (#15,596,018, Invitrogen) according to the manufacturer’s instructions. RNA was reverse-transcribed to cDNA using HiScript® III RT SuperMix for qPCR (+ gDNA wiper) (#R323-01, Vazyme), and quantitative PCR was performed using the ChamQTM SYBR® qPCR Master Mix (#Q321-02, Vazyme) in a Roche LightCycler 480 System (Roche). PCR conditions were as follows: initial denaturation for 15 s at 95 °C, followed by 40 cycles of 10 s at 95 °C, and 30 s at 60 °C. GADPH was used as the housekeeping gene for mouse samples. Gene expression was calculated using the 2^−ΔΔCt^ method. All primers used are listed in Additional file 1: Table S2, and were synthesised by Tsingke Biotechnology Co., Ltd.

### Statistical analysis

All values are expressed as the mean ± SEM (standard error of mean). Statistical analysis was performed using the GraphPad Prism 9.0 software (GraphPad Software, La Jolla, CA, USA). Comparisons between two groups were either performed by two-tailed Student’s *t*-test in the case of normal distribution or Mann–Whitney U test if the data were not normally distributed. One-way ANOVA was used for comparisons between more than two groups. The level of statistical significance was set at *p* value < 0.05.

## Results

### Infiltration and activation of MCs are increased in psoriatic lesioned skin

**Fig. 1:**
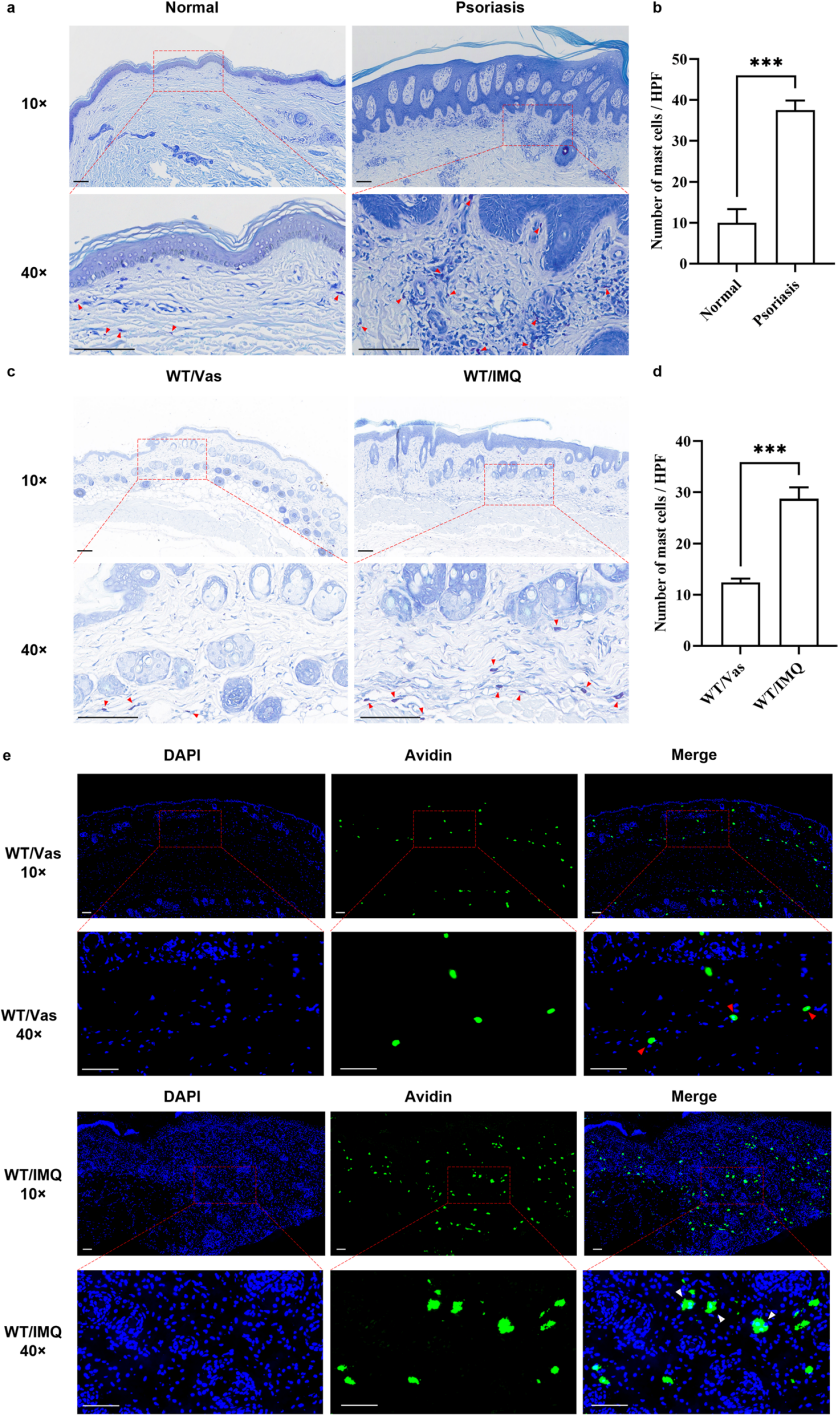
Mast cells increase in psoriatic lesions. **a** and **b** Representative images of toluidine blue staining in paraffin skin sections obtained from healthy controls and patients with psoriasis (n = 4). **c** Representative images of toluidine blue staining in paraffin skin sections from control and IMQ-treated mice (n = 4). Red arrows indicate MCs. **d** Bar graph shows MCs quantitation between the two groups. Data are represented by mean ± SEM; * *p* < 0.05, ** *p* < 0.01, and *** *p* < 0.001. **e** Representative images of avidin immunostaining in paraffin skin sections from control and IMQ-treated mice. Red arrows indicate non-degranulated MCs, white arrows indicate degranulated MCs. Scale bar = 100 μm

We first examined the number of MCs in skin biopsies using toluidine blue staining. The number of MCs was significantly increased in the psoriatic lesions compared to that in the normal skin controls (shown in Fig. 1a and b). A similar increase in the number of MCs was also observed in the skin of the IMQ-induced psoriasis-like mice (shown in Fig. 1c and d).

To further evaluate the activation of mast cells, we performed histological staining with avidin that specifically binds to MC granules [18]. In contrast to the typical round or oval morphology of MCs observed in the dermis of Vaseline control mice, MCs in the dermis of IMQ-induced psoriasis-like mice exhibited distinctive characteristics (shown in Fig. 1e). The cell membranes of MCs are discontinuous with a partial loss of the typical round or oval shape of MCs; the size of the cells increased; granular residues were visible around MCs; some MCs appeared as a conglomeration of degranulated fragments [19]. Overall, these data confirm the infiltration and activation of MCs in psoriasis, suggesting their possible role in the immune regulation of psoriasis.

### MCs deficiency ameliorates IMQ-induced psoriasis-like dermatitis in mice at an early stage

**Fig. 2:**
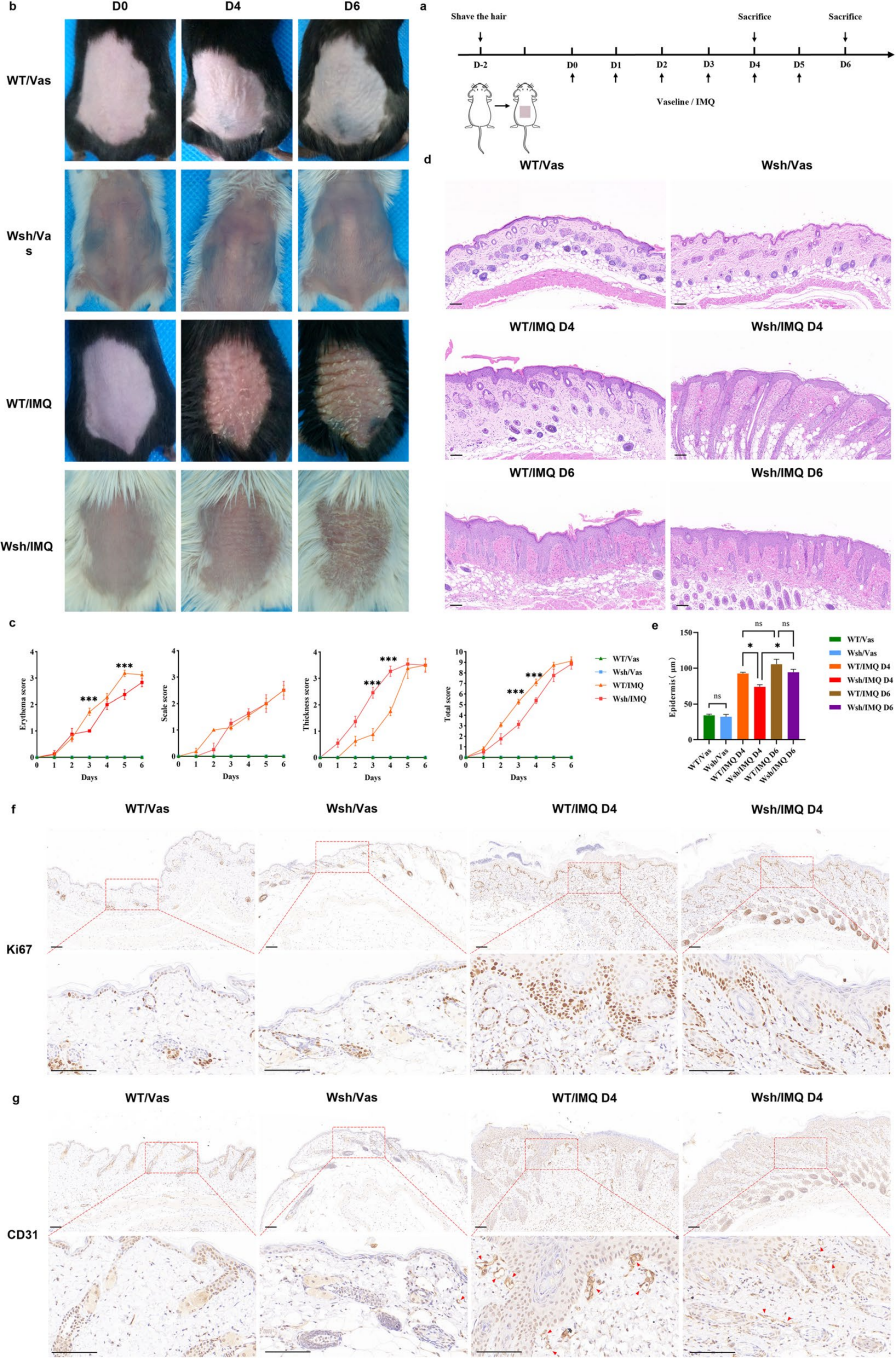
Impact of Mast cells on psoriasis development. **a** Schematic representation of the animal study design, namely IMQ or Vaseline administration timepoints. **b** Representative photographs of the lesioned skin of mice from each group on Days 0, 4 and 6. **c** Disease severity scoring of each group, based on scaling, erythema, and skin thickness, from Day 0 to Day 6. **d** Representative haematoxylin & eosin staining of skin sections of mice from each group on Days 4 and 6. **e** Epidermal thickness measurements of skin sections for each group (n = 3). Scale bar = 100 μm. Results are representative of three independent experiments. Data are represented by mean ± SEM; * *p* < 0.05, ** *p* < 0.01, and *** *p* < 0.001. **f** and **g** Representative photomicrographs (10 × and 40 × magnification) of skin sections of mice from each group on Day 4, stained with ki67 (**f**) and CD31 (**g**). Red arrows indicate CD31^+^ vessels (brown). Scale bar = 100 μm

To investigate the relevance of MCs for IMQ-induced psoriasis-like dermatitis, we topically applied IMQ to the dorsal skin of Kit^Wsh/Wsh^ mice and their wild-type (WT) littermates for six consecutive days to construct a psoriasis-like mouse model (shown in Fig. 2a). Compared to WT, a significant alleviation of the severity of skin inflammation, including erythema, scaling, and thickness, was observed in Kit^Wsh/Wsh^ mice during the preliminary phase, with the most significant differences on Days 3 and 4 (*p* < 0.001). At Day 6, there were no differences between both groups (shown in Fig. 2b–e).

Subsequently, we studied keratinocyte proliferation using Ki67 staining (a proliferation marker) and assessed neovascularisation by CD31 staining. Compared to WT mice, Kit^Wsh/Wsh^ mice showed significantly reduced keratinocyte proliferation, lower vessel density, and smaller vessel diameter in the early stages of IMQ-induced psoriasis-like dermatitis (shown in Fig. 2f and g). These results suggest that MCs play a substantial role in the promotion of the early stages of psoriatic dermatitis.

### MCs influence the immune and inflammatory response pathways in IMQ-induced psoriasis-like dermatitis

**Fig. 3:**
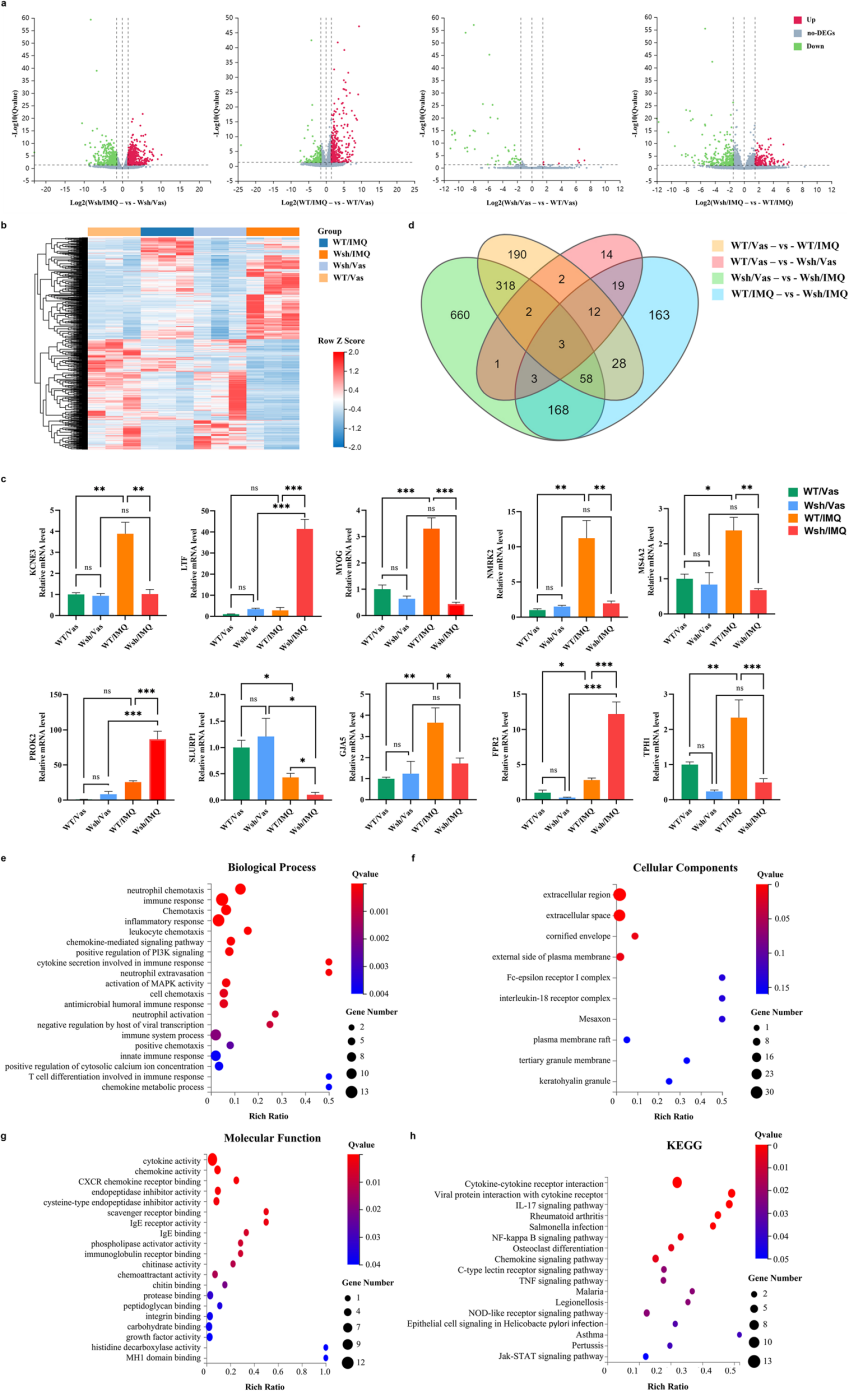
Altered transcriptome in IQM-induced psoriasis mouse models. Kit^wsh/wsh^ mice (n = 3) and their littermate controls (n = 3) were treated with IMQ and Vaseline topically for four consecutive days. Skin samples were collected on Day 5 for transcriptome analysis. **a** Volcano plot of differentially expressed genes (DEGs) in each group (|log_2_FC|≥ 1.5 and *p* ≤ 0.05; red: up-regulated DEGs; green: down-regulated DEGs). **b** Hierarchical Clustering analysis of DEGs (red: high relative expression; blue: low relative expression). Expression levels were subjected to Z score scaling within each sample for visualisation purposes. **c** Gene expression levels of KCNE3, LTF, MYOG, NMRK2, MS4A2, PROK2, SLURP1, GJA5, FPR2, and TPH1 were measured using real-time PCR. Data were normalised to GADPH expression levels and reported in relative expression units. Data are represented by mean ± SEM from three independent experiments. * *p* < 0.05, ** *p* < 0.01, and *** *p* < 0.001. **d** Venn diagram of the overlapping DEGs from the four experimental groups (WT/Vas vs WT/IMQ; WT/ Vas vs Wsh/ Vas; Wsh/ Vas vs Wsh/IMQ; WT/IMQ vs Wsh/IMQ). **e–g** GO enrichment analysis of DEGs in biological process (**e**), cellular components (**f**), and molecular function (**g**). **h** KEGG pathway enrichment analysis of the DEGs

To elucidate the molecular mechanisms underlying the association of MCs with psoriasis development at an early stage, we performed RNA-seq analysis of back skin lesions collected from Kit^Wsh/Wsh^ and WT mice after the 4-day treatment with IMQ and Vaseline. DESeq software was used for the analysis of differential gene expression to identify candidate genes regulated by MCs in psoriasis. A total of 2336 DEGs were identified in comparisons between the four groups (shown in Additional file 2: Table S3). A paired comparison between the transcriptomes of healthy and psoriatic-lesioned skin revealed 613 DEGs, of which 346 were up-regulated and 267 down-regulated. The comparison of the transcriptomes of healthy and psoriatic-lesioned skin from MC-deficient mice identified 1213 DEGs, of which 624 genes were up-regulated and 589 were down-regulated. Only 56 DEGs were uncovered upon comparing the skin of the two control groups, WT/ Vas and Wsh/ Vas, suggesting a similar skin transcriptomic profile under the physiological state. However, the comparison between the two mouse models post-stimulation with IMQ revealed 454 DEGs, with 148 up-regulated and 306 down-regulated (shown in Table 1 and Fig. 3a). Unsupervised hierarchical clustering of DEGs clearly discriminated between the subgroups (shown in Fig. 3b). RNA-sequencing results were confirmed using real-time PCR; 10 DEGs were randomly selected for testing, and the results were consistent with the sequencing results (shown in Fig. 3c).

**Table 1:** Number of differentially expressed genes in each of the comparisons

	Number of genes		
DE analysis	DEGs	Up-regulated	Down-regulated
WT/Vas versus WT/IMQ	613	346	267
WT/Vas versus Wsh/Vas	56	7	49
Wsh/Vas versus Wsh/IMQ	1213	624	589
WT/IMQ versus Wsh/IMQ	454	148	306

A Venn diagram of these DEGs was generated to depict the results in each subgroup (shown in Fig. 3D). We focused our subsequent analyses on a total of 86 genes from the “WT/IMQ *vs.* Wsh/IMQ” condition that overlapped with those of the “WT/Vas *vs.* WT/IMQ” condition, after excluding the DEGs representing the physiological functions of the MCs (uncovered in the “WT/Vas *vs.* Wsh/Vas” comparison) (shown in Fig. 3d). The biological characteristics of the potential targets of MCs in psoriasis were evaluated using GO and KEGG-based enrichment analyses. GO analysis is widely used to classify gene products according to three categories, namely biological processes, cellular components, and molecular functions. For the overlapping DEGs, the major significant emerging terms were neutrophil chemotaxis, immune response, and chemotaxis for biological processes; extracellular region, extracellular space, and cornified envelope for cellular components; and cytokine activity, chemokine activity, and CXCR chemokine receptor binding for molecular functions (shown in Fig. 3e–g). In addition, significant KEGG pathways were associated with cytokine–cytokine receptor interaction, viral protein interaction with cytokine receptors, and the IL-17 signalling pathway (shown in Fig. 3h). In summary, the RNA-seq analyses revealed the heterogeneity and complexity of MC-related genes during psoriasis-like disease initiation and progression.

### IL-33 exacerbates IMQ-induced psoriasis-like dermatitis, partly through MC activation

**Fig. 4:**
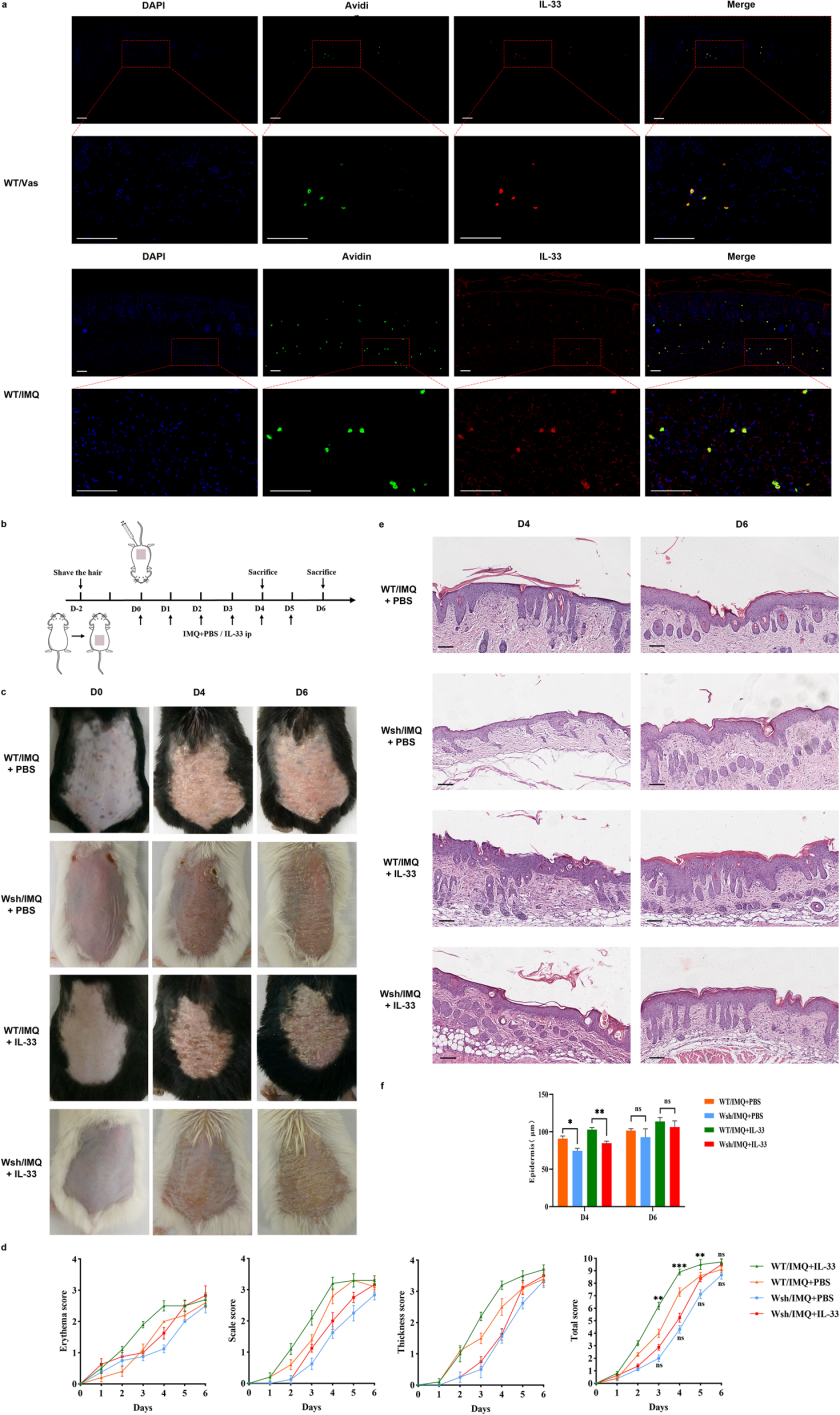
Effect of IL-33 administration on MC-mediated IMQ-induced psoriasis-like dermatitis. **a** Skin lesion tissues of IMQ-induced WT mice and Vaseline controls co-labelled for IL-33 (red) and Avidin (green). Merged image shows co-localisation of the two markers (yellow). Sections were counterstained with DAPI (blue) for nuclear staining. Scale bar = 100 μm. **b** Schematic representation of the protocol design for the treatment with recombinant mouse IL-33 and IMQ. **c** Representative photographs of the lesioned skin of mice from each group on Days 0, 4 and 6. **d** Disease severity scoring of each group, based on scaling, erythema, and skin thickness, from Day 0 to Day 6. The upper marker represents the results of WT/IMQ-PBS *vs.* WT/IMQ-IL-33, and the lower one represents the results of Wsh/IMQ-PBS *vs*. Wsh/IMQ-IL-33. **e** Representative haematoxylin & eosin staining of skin sections of mice from each group on Days 4 and 6. Scale bar = 100 μm. **f** Epidermal thickness measurements of skin sections for each group (n = 3). Results shown are representative of three independent experiments. Data are represented by mean ± SEM; * *p* < 0.05, ** *p* < 0.01, and *** *p* < 0.001

The mechanism underlying the impact of MCs on psoriasis appears to be highly correlated with the interaction of cytokines and cytokine receptors. Considering that MCs constitutively express IL-33R, IL-33 is a potent MC activator, and IL-33 has been shown to play a critical regulatory role in psoriasis, we hypothesised that IL-33 could promote activation of MCs and thus regulate psoriasis development. To verify this, we first confirmed, using immunofluorescence, whether IMQ topical application could increase IL-33 expression in the skin lesions, which was significantly elevated in the dermis in addition to keratinocytes. Immunofluorescent co-labelling for IL-33 and avidin showed an overlap between these two markers in the dermis (shown in Fig. 4a).

To analyse the role of IL-33-MC interaction in psoriasis, we investigated the effect of exogenous IL-33 on the development of IMQ-induced psoriasis under MC-deficient conditions. IMQ was applied topically to Kit^Wsh/Wsh^ mice and WT mice for 6 days, and each mouse was injected intraperitoneally with recombinant murine IL-33 (1 µg/100 μL) or PBS for six consecutive days (shown in Fig. 4b). In WT mice, IL-33 significantly exacerbated IMQ-induced psoriasis-like dermatitis compared to the control mice. However, in Kit^wsh/wsh^ mice, the exacerbating effect of IL-33 was blocked, but skin inflammation severity scores in this group increased from Day 5 compared to the control group, although without statistical significance. In addition, exogenous IL-33-treated Kit^wsh/wsh^ mice showed a significant relief of IMQ-induced epidermal thickening at an early stage compared to WT mice and converged to similar levels as those of control mice by Day 6 (shown in Fig. 4c–f). Together, these data suggest that IL-33 partially mediates the exacerbating effect of MCs on psoriasis.

## Discussion/conclusion

MCs have not been a major focus of psoriasis research, and results from multiple studies have been inconsistent. In this study, we investigated the role of MCs in psoriasis development. We identified an upregulation in MC infiltration and activated degranulation in skin lesions from patients with psoriasis and IMQ-induced psoriasis-like dermatitis. Using MC-deficient Kit^wsh/wsh^ mice, we observed that MCs are responsible for an early exacerbation of the IMQ-induced psoriasis-like dermatitis response, which is highly correlated with the cell-cytokine receptor interaction pathway. We further demonstrated that the level of IL-33 is upregulated and co-localised with MCs in the psoriatic dermis and that MC-deficient mice show a delayed response to exogenous IL-33-exacerbated IMQ-induced psoriasis-like dermatitis. Hence, our results identify MCs as a promising target for psoriasis therapeutic strategies.

MCs play a crucial role in host defence against harmful pathogens, but paradoxically, they are also involved in the pathogenesis of inflammatory diseases [20]. The mechanisms by which MCs function may vary considerably across diseases. When activated through degranulation, MCs release numerous pre-formed and pre-activated immunomodulatory compounds into the extracellular environment, accounting for both the protective and detrimental effects of MCs in various inflammatory settings [21]. This inflammation-modulating effect has been described for a variety of diseases [22, 23]. The uptake of exogenous MC granules boosts dendritic cell (DC) activation and T-cell priming capacity, exacerbating lipopolysaccharides (LPS) -driven skin inflammation [24]. In contrast, in ultraviolet B-induced skin damage, MCs limit the development of skin inflammation through IL-10 [25].

Considering the bidirectional nature of MCs’ immunomodulatory functions and the lack of clarity regarding the specific role of MCs in psoriasis, we used MC-deficient Kit^wsh/wsh^ mice to better understand the connection between MCs and disease development. We observed that MCs exacerbate IMQ-induced psoriasis-like dermatitis, as demonstrated using CD31 and Ki67 immunostaining; however, this effect was only differential in the early stages of disease development. Other studies have reported that IMQ-induced psoriasis-like mice show a biphasic presentation. Application of IMQ to the dorsal skin for 14 consecutive days revealed a consistent increase in clinical parameters such as skin thickness, erythema, and desquamation in the early phase, which reached a plateau on Day 7. During the late phase (Days 8–14), these clinical parameters remained stable [26]. Considering that the IMQ-induced psoriasis model showed self-remission after 5–6 days, in this experiment, we defined the period up to Day 5 of IMQ application as the early stage for the follow-up study.

In agreement with other studies [27], avidin staining of IMQ-treated skin showed that MCs were significantly activated for degranulation. The MC number and degranulation in early psoriatic lesions were reported to be higher than those in mature psoriatic lesions [28]. Notably, it is believed that there exists a bimodal immune activation phenomenon in psoriasis, with innate immune cells such as neutrophils, activated MCs, and plasmacytoid DCs predominating in an early stage of psoriasis and T lymphocytes and monocytes characterising advanced, stable psoriatic lesions [29]. MCs can initiate the early stages of neutrophil recruitment [30–33]. IgE-independent activated MCs have the unique ability to secrete pre-formed TNF-α, which is a prerequisite for neutrophil recruitment to inflamed skin [34]. MCs are the closest to the vascular system, initiating the early stages of neutrophil recruitment by releasing the chemokine CXCL1/CXCL2, 1 h after LPS stimulation. Upon reaching the tissue, neutrophils further infiltrate in a macrophage-dependent manner [30]. This could possibly explain why the role of MCs was not as evident at later time points, as the redundant role of other immune cells might maintain psoriasis development.

Until recently, research predominantly focused on IgE-mediated MC activation; however, novel MC activation paradigms, independent of IgE and with unique cytokine secretion profiles, have received increasing attention. MCs have several types of receptors on their surface, including cytokine receptors [35]. In this study, DEGs in IMQ-induced skin lesions in Kit^wsh/wsh^ mice were enriched in the cytokine-cytokine receptor interaction pathway. In addition to releasing cytokines themselves, MCs can also be activated by exogenous cytokines via cytokine receptors on their surfaces. One of the most widely discussed pathways is mediated through IL-33/ST2 signalling [3]. The IL-1 cytokine family, including IL-33, is a critical regulator of inflammation. Generally released rapidly upon external stimulation and subsequently acting as an alarm cytokine to mediate immune responses, IL-33 exhibits a dual functional immunomodulatory role in several diseases, which may depend on its primary target cells, on IL-33/sST2 expression levels, cellular context, and the cytokine microenvironment [7, 36]. In psoriasis, IL-33 expression is increased in patient lesions and is thought to be released primarily by keratinocytes [11]. Consistently, we observed significantly elevated IL-33 expression in the epidermis of IMQ-induced psoriasis-like lesions. Furthermore, we observed that a significantly enhanced IL-33 signal was present in the dermis and significantly overlapped with the avidin-stained MCs. This suggests that MCs are possibly the target cells for the IL-33 released by keratinocytes. Brief exposure to IL-33 enhances ATP-induced MCs cytokine production above the threshold, for inflammation initiation [37, 38]. In addition, IL-33 enhances the effect of substance P (SP) on VEGF released from MCs and activates the secretion of CXCL2 by MCs, which increases vascular permeability and initiates neutrophil infiltration [4, 39].

Our study further revealed that IMQ-induced Kit^Wsh/Wsh^ mice showed a delayed response to exogenous IL-33 compared to WT mice, manifesting as the masked alleviation of MC deficiency at the late stage of exogenous IL-33 administration. A possible explanation for this phenomenon is the difference between long- and short-term IL-33 effects. An acute burst of IL-33 supports degranulation to promote MC-dependent defence strategies. Conversely, MCs convey protection against overt and persistent IL-33 by shutting down degranulation, thus avoiding exaggerated responses and restoring tissue homeostasis under chronic inflammatory conditions [40, 41]. Further studies on MCs and IL-33 levels in the functional dynamics of skin lesions of acute and chronic phase psoriasis patients, and in constructed long-term psoriasis models, may help elucidate their specific role in disease development.

In conclusion, these findings provide new insights into the role of MCs in psoriasis. Activation of MCs by the alarmin IL-33, released from stimulated keratinocytes, promotes the early initiation of inflammatory and immune responses in this disease. In psoriasis pathogenesis, MCs and IL-33 may strike a delicate balance between pro- and anti-inflammatory functions, playing a role as immunomodulatory or homeostatic factors. The interaction between IL-33 and MCs further reiterates the impact of the innate immune network in psoriasis, suggesting that two-stage combination therapies aimed at suppressing innate and adaptive immune responses may be a substantial strategy for psoriasis treatment.

## Supplementary Information

**Additional file 1. Table S1.** Demographics of patients whose skin samples were collected and **Table S2** Primers for real-time PCR analysis.

**Additional file 2. Table S3.** Differentially expressed genes

## Data Availability

All data generated or analysed during this study are included in this article and its online supplementary material. Further inquiries can be directed to the corresponding author.

## Abbreviations

MCs: Mast cells
IL-33R: Interleukin-33 receptor T1/ST2
WT: Wild-type
IMQ: Imiquimod
CRC: Colorectal cancer
Vas: Vaseline
PSI: Psoriasis severity index
H&E: Haematoxylin and eosin
IHC: Immunohistochemistry
BSA: Bovine serum albumin
DEGs: Differentially expressed genes
GO: Gene Ontology
KEGG: Kyoto Encyclopaedia of Genes and Genomes
DC: Dendritic cell
LPS: Lipopolysaccharides
