# Supplementary material for: IL-33-mediated activation of mast cells is involved in the progression of imiquimod-induced psoriasis-like dermatitis
Source: Cell Commun Signal. 2023 Mar 9;21:52. doi: 10.1186/s12964-023-01075-7 (PMC9996901; doi:10.1186/s12964-023-01075-7)
Supplement: Supplementary file 2 — Additional file 1. Table S1. Demographics of patients whose skin samples were collected and Table S2 Primers for real-time PCR analysis. [file 12964_2023_1075_MOESM2_ESM.docx]

**Supplementary Materials**

**Table S1 Demographics of patients whose skin samples were collected**

| **Patient Category** | **Sample ID** | **Age** | **Gender** | **Location of Skin Tissues** |
| --- | --- | --- | --- | --- |
| Normal skin (patients with No psoriasis) | #1 | 30 | Male | Back |
|  | #2 | 42 | Male | Back |
|  | #3 | 67 | Male | Abdomen |
|  | #4 | 53 | Female | Thigh |
| Psoriatic skin | #1 | 29 | Male | Thigh |
|  | #2 | 57 | Female | Back |
|  | #3 | 43 | Male | Abdomen |
|  | #4 | 73 | Male | Abdomen |

Abbreviation: ID, identification.

**Table S2 Primers for real-time PCR analysis**

| **RNA** | **Primer Sequence (5′–3′)** | |
| --- | --- | --- |
| mKCNE3 | Forward | ATGCTGTGCTGAAGGCTCTGAAC |
|  | Reverse | GGAGTTGTCATTACGACCAGGAAGG |
| mLTF | Forward | TGGCTTGGATTGTGTGAACAGACC |
|  | Reverse | GGCAGTGTGGCAGGACTTCTTG |
| mMYOG | Forward | GACAGCATCACGGTGGAGGATATG |
|  | Reverse | CACACCCAGCCTGACAGACAATC |
| mNMRK2 | Forward | GACCGTGCCGTATGAGGAATGC |
|  | Reverse | GTCCTGCTCCATCTCCCGTCTATAC |
| mMS4A2 | Forward | TGCTCTCCCAAATCCACAAGAATCC |
|  | Reverse | GCTCCCAGGAACTCCAACTCTTTC |
| mPROK2 | Forward | CTGCTACCGCTGCTGTTCACAC |
|  | Reverse | TCCTCCGCACTGAGAGTCCTTG |
| mSLURP1 | Forward | GGAGACAGTGGAAGCAGCGTTC |
|  | Reverse | CGGAAGCAACAGAAGACAGGATGG |
| mGJA5 | Forward | CATGCACTATAGCCAGAAGCCAGAG |
|  | Reverse | TGTCACTATGGTAGCCCTGAGGAAG |
| mFPR2 | Forward | GATCCTCTCAATGGTGGTTGTCTCC |
|  | Reverse | GTGGTGACAGTGTGTGGCATCC |
| mTPH1 | Forward | ATCCGTCCTGTGGCTGGTTACC |
|  | Reverse | AGGTGTCTGGCTCTGGAGTGTAG |
